# Sequence analysis and plasmid mobilization of a 6.6-kb kanamycin resistance plasmid, pSNC3-Kan, from a *Salmonella enterica* serotype Newport isolate

**DOI:** 10.1371/journal.pone.0268502

**Published:** 2022-07-14

**Authors:** Chin-Yi Chen, Ly-Huong T. Nguyen, Terence P. Strobaugh

**Affiliations:** Molecular Characterization of Foodborne Pathogens Research Unit, Eastern Regional Research Center, Agricultural Research Service, U.S. Department of Agriculture, Wyndmoor, Pennsylvania, United States of America; University of Graz, AUSTRIA

## Abstract

Research on the transfer of antibiotic resistance plasmids has been mainly focused on the large multi-drug resistance conjugative plasmids, while the transmission of small mobilizable plasmids remains under-investigated. A series of diverse ColE-like kanamycin resistance plasmids (“KanR plasmids”) from *Salmonella enterica* were characterized previously. In this study, the 6.6-kb pSNC3-Kan from a *Salmonella enterica* serotype Newport isolate was investigated. It possessed highly conserved RNA I/II and Tn*602* (IS*903*-*aph*-IS*903*) regions to two other KanR plasmids pSe-Kan and pSBardo-Kan, but carried a *mobC-mobA/BD* operon. The mobilization proteins encoded by the *mob* operon of pSNC3-Kan showed high sequence identity (~95%) to those of an *E*. *coli* plasmid pEC34B, except that MobE was not present; and were much less conserved to those of another KanR plasmid pSN11/00Kan (43% - 86% identity). Four structurally different KanR plasmids were investigated for their ability to be mobilized by the conjugal transfer (*tra*) genes from F and IncP plasmids. Transfer genes derived from IncP plasmids can efficiently mobilize KanR plasmids possessing the *mob* operons (*mobC-mobA/BD*), such as pSNC3-Kan and pSN11/00Kan, in bi-parental mating experiments. On the other hand, F *tra* genes were able to mobilize pU302S, pSNC3-Kan and pSe-Kan, but not pSN11/00Kan. A plasmid-borne *mob* operon was not required for mobilization of the *oriT(F)*-bearing pSe-Kan by the F *tra* genes. This study underscores the complexity of plasmid interaction and the importance of how small mobilizable plasmids may contribute to the spread of antibiotic resistance genes.

## Introduction

Outbreaks involving *Salmonella enterica* accounted for 30% of foodborne diseases of known etiology that occurred between 2009–2015 in the United States [1]. They are responsible for an estimated 1.2 million annual salmonellosis cases just in the U.S. alone and account for both leading causes of hospitalization and deaths among the major foodborne pathogens [2, 3]. More alarmingly, there was increased resistance to third-generation drugs such as cephalosporins, fluoroquinolones, or azithromycin as reported in the 2016–2017 National Antimicrobial Resistance Monitoring System (NARMS) report [4]. *S*. *enterica* serotype Newport is the third most common serotype in human salmonellosis where a recent study showed that 8% of Newport isolates collected were resistant to seven antibiotics, including ceftriaxone [5]. This is a cause for concern because the resistance genes may be acquired by other bacteria through bacteriophage or by other horizontal gene transfer events where plasmids play a key role [6].

In addition to the larger (generally > 60-kb) conjugative plasmids, smaller mobilizable plasmids, although not self-transmissible, can be transferred between bacteria by the transfer (*tra*) machinery of conjugative plasmids. These small plasmids are usually under 15-kb, present in high copy numbers and may only carry minimal gene sets, yet provide an efficient means for gene spread in bacterial populations [7]. These mobilizable plasmids generally contain an origin of transfer (*ori*T), and encode a relaxase, and/or other mobilization accessory proteins, which are required to interact with the type IV secretion system (T4SS) to help with the transfer process [8, 9], although exceptions do exist (see “Results and discussion” section below). Accessory DNA binding proteins containing the ribbon-helix-helix (RHH) domain are involved in specific sequence recognition and binding near the *nic* site to help relaxase to form relaxosome and stimulate nickase function [9]. One notable example is the ColE1 plasmid, which possesses *mobC-mobA/BDE* genes and can be mobilized by conjugative plasmids of different incompatibility groups (IncF, IncP, IncI, and IncW) [10, 11]. The ColE superfamily of the mobilizable plasmids can be further divided into MOB-HEN and MOB-P subfamilies with different mobilization gene structures [8]; the classification has been further redefined in recent years and the ColE family now are grouped into the MOB_P5_ family based on their relaxases [12, 13]. Other ColE-like (“Col”) plasmids were shown to lack mobilization relaxase. These may utilize a conserved protein that shares ~50% identity to the NikA relaxase accessory protein of IncI1 plasmid R64, which is distantly related to MobC, to interact with the relaxase and T4SS of different conjugative plasmids (such as R64) to facilitate transfer [14]. MobC/MbeC and NikA on the mobilizable plasmids are RHH proteins that serve similar functions as the TraM and TraY proteins of the F conjugative system. TraM and TraY are multi-domain RHH DNA-binding proteins that bind to multiple sites within the F *oriT* and interact with other conjugative proteins and host factors. TraY and IHF (integration host factor) interact with TraI relaxase, cause DNA-bending and stimulate the nicking reaction of TraI. TraM, although not required for cleavage, was shown to be important in bringing the relaxasome complex to the mating pore by interacting with TraD coupling protein, and that TraM-DNA interaction determines plasmid specificity (for reviews, see [9, 15]).

In previous studies we identified several dozens of kanamycin resistance ColE1-like plasmids (referred to as “KanR plasmids” hereafter) from *Salmonella enterica* isolates collected by NARMS and categorized them into five different groups (A, B, C, X and Y) based on their restriction digest patterns [16, 17]. Aside from all carrying an *aph(3’)-I* gene (encoding the APH(3’)-I family aminoglycoside O-phosphotransferase), each KanR plasmid group showed distinct structure/features such as RNA I/II, mobilization genes, origins of transfer (*oriT*), and IS elements [18–20]. The *aph(3’)-I* allele on the group C/C2/C3 KanR plasmids encodes a protein differed by four residues to those of the groups A and B plasmids [16]. Plasmids pSe-Kan (group C) and pSBardo-Kan (group C2) were sequenced, and the *aph(3’)-I* gene was shown to be flanked by two copies of IS*903*s and lack the *mobC*-*mobA/BD* operon typical of the ColE-like plasmids [20]. pSBardo-Kan possessed an extra copy of IS*903*, but is otherwise nearly identical to pSe-Kan [20]. A third member (designated as C3) that showed 100% identity in the RNA I/II region and the *aph(3’)-I* gene to the C/C2 plasmids, but with differences in the restriction digest patterns, remained uncharacterized. To further understand the diversity of these KanR plasmids, we present here the sequence analysis of the pSNC3-Kan plasmid from *S*. Newport strain ARS #574, and evaluation of the mobilization of four representative KanR plasmids by F and IncP conjugal transfer systems.

## Materials and methods

### Plasmid sequencing and analyses

Plasmid DNA from the DH5α transformant carrying the small KanR plasmid derived from *S*. Newport isolate ARS #574 [16] was purified using a QIAGEN Plasmid Midi kit (QIAGEN). Sanger sequencing was performed using a dye terminator reaction (BigDye terminator v. 3.1; Invitrogen) with custom primers that were designed based on previously sequenced plasmids [20], as well as pSNC3-Kan, and ran on an ABI 3730 sequencer (Applied Biosystems). Sequences were assembled and circularized using Sequencher (v. 5.0, Gene Codes Corp., Ann Arbor, MI). Preliminary ORF identification and annotation was performed using NEBCutter (v.2.0; http://nc2.neb.com/NEBcutter2/; [21]). Plasmid maps were generated using MacVector (v. 15.5.4). Geneious (R11.0.3; Biomatters, Ltd.) was used for data curation and bioinformatics analysis such as amino acid translation and sequence alignment. BLAST (NCBI, https://blast.ncbi.nlm.nih.gov/Blast.cgi; [22–24]) was used for sequence homology search and comparison. Identification of plasmid replicon and origin of transfer (*oriT*) were conducted using PlasmidFinder v2.1 (https://cge.cbs.dtu.dk/services/PlasmidFinder/; [25]) and *oriT*Finder (http://bioinfo-mml.sjtu.edu.cn/oriTfinder/; [26]), respectively. Default settings were applied unless specified. Phylogenetic analysis of the complete nucleotide sequence of the KanR plasmids and other plasmids utilized MUSCLE multiple sequence alignment [v3.8.425; 27] on the EMBL-EBI website (https://www.ebi.ac.uk; [28]) using the default setting and output (Neighbor-joining tree without distance correction).

### KanR plasmid mobilization experiments

Detailed information on the bacterial strains and plasmids used in this study are listed in Table 1. To generate the donor strains for bi-parental mating: KanR plasmids (purified from DH5α host) were transformed into NEB5αF’I^q^ (carrying F’; NEB; library-efficiency competent cells) or S17-1λ*pir* (RP4/IncP *tra* gene integrated into the chromosome; gift from Dr. Clay Fuqua, Indiana University, Bloomington, IN; [29]). Mating procedure was modified from that used by Brasch and Meyer [30]. Briefly, cells were grown overnight at 37°C in LB with required antibiotic. On the day of mating, cells were sub-cultured in LB without antibiotics and grown to mid-log phase (OD_600_ ~0.3–0.4); cells were then washed, resuspended in LB, and spotted on a LB agar plate at 1:10 (donor: recipient) ratio. Mixtures were allowed to dry at room temperature briefly (~15 min), and then incubated at 37°C for 60 min. Cells were collected from the mating plate, resuspended in LB, and serially diluted and plated on LB agar supplemented with antibiotics using 6×6 drop plate methods [31]. Donor strain S17-1λ*pir* carrying KanR plasmid was paired with recipient CAG18483 (Tet^R^) and transconjugants were selected on LB supplemented with Kan 50 μg/mL and Tet 2 μg/mL. NEB5αF’I^q^ donor strain carrying KanR plasmid was paired with recipient NEB10β (Str^R^) and transconjugants selected on LB supplemented with Kan 50 μg/mL and Str 50 μg/mL. The proportion of the transconjugants carrying mobilized KanR plasmid (Kan^R^Str^R^ or Kan^R^Tet^R^) in the population of kanamycin resistant bacteria was calculated to evaluate the mobilization capability by different *tra* systems. Total Kan^R^ cells include transconjugants and donors. Average and standard deviation were calculated from results of three or more experiments. For NEB5αF’I^q^ x NEB10β experiments, random transconjugant colonies (n = 3–8) were picked from the most diluted spots on the selective plates (without further culturing) for plasmid miniprep using QIAprep spin miniprep kit (QIAGEN). Plasmid preps were digested with *Xba*I + *Hin*dIII and resolved on 1% TAE-agarose gel to verify the presence of mobilized KanR plasmids.

**Table 1:** Bacterial strains and plasmids used in this study.

Name	Note	Relevant features	Source or GenBank accession no.
**Strain**			
DH5α	KanR plasmid propagation	F^-^ *Δ(argF-lacZ)U169 phoA glnV44 U80 Δ(lacZ)M15 gyrA96 recA1 relA1 endA1 thi-1 hsdR17*	Lab collection
NEB5αF’I^q^	Biparental mating donor (F *tra*)	Tet^R^; F’ *proA*^*+*^*B*^*+*^* lacI*^*q*^* Δ(lacZ)M15 zzf*::*Tn10 *(Tet^R^)* / fhuA2Δ(argF-lacZ)U169 phoA glnV44 Φ80Δ(lacZ)M15 gyrA96 recA1 relA1 endA1 thi-1 hsdR17*	New England BioLabs
NEB10β	Bi-parental mating recipient	Str^R^; F^-^ Δ*(ara-leu)7697 araD139 fhuA* Δ*lacX74 galK16 galE15 e14-Φ80Δ(lacZ)*Δ*M15 recA1 relA1 endA1 nupG rpsL *(Str^R^)* rph spoT1 *Δ*(mrr-hsdRMS-mcrBC)*; paired with NEB5αF’I^q^	New England BioLabs
S17-1λ*pir*	Biparental mating donor (IncP *tra*)	*RP4-2(Km*::*Tn7*,*Tc*::*Mu-1) pro-82 λpir recA1 endA1 thiE1 hsdR17 creC510*	Clay Fuqua [29]
CAG18483	Biparental mating recipient	Tet^R^; F^-^, *fad*L771:Tn10; paired with S17-1λ*pir*	Coli Genetic Stock Center; CGSC#7407
**Plasmid**			
pU302S	3208 bp; group A KanR plasmid	Kan^R^; *nikA*, *oriT*(pEC34A-type); from *Salmonella enterica* serovar Typhimurium strain G8430	AY333433 [18]
pSN11/00Kan	5698 bp; group B KanR plasmid	Kan^R^; *mobC-mobA/BD*, *oriT*(ColE-type); from *Salmonella enterica* serovar Newport strain SN11/00	GQ470395 [19]
pSe-Kan	7132 bp; group C KanR plasmid	Kan^R^; *oriT*1(ColE-type), *oriT*(F-type), no *mob*; from *Salmonella enterica* serovar Typhimurium DT104 strain ARS# 852	HQ230976 [20]
pSNC3-Kan	6606 bp; group C3 KanR plasmid	Kan^R^; *mobC-mobA/BD*, *oriT*(ColE-type); from *Salmonella enterica* serovar Newport strain ARS# 574	MW030687 (this study)

## Results and discussion

### Sequence features of pSNC3-Kan

**Fig 1:**
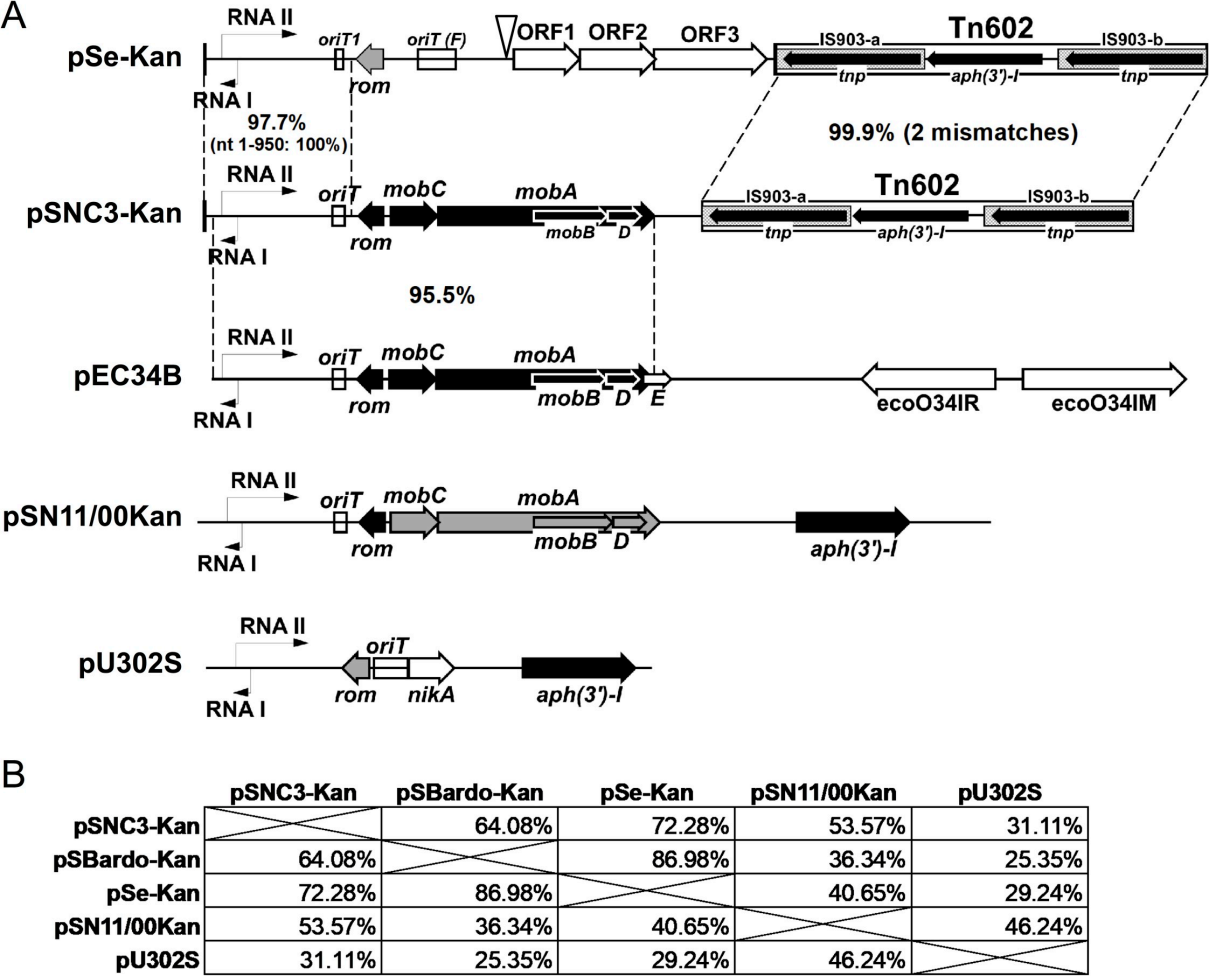
KanR plasmid maps and comparisons. (A) Schematic comparison of pSNC3-Kan to pSe-Kan and pEC34B. Plasmid regions sharing homologies with pSNC3-Kan are connected by dashed lines and the % nucleotide identity indicated. Maps of pU302S and pSN11/00Kan are also included. RNA I/II are indicated by thin lines with an arrow head; *oriT* regions are indicated by open boxes; IS elements are shown as patterned boxes with an internal arrow indicating the *tnp* ORF; target site duplications are shown as thick vertical line bordering the Tn*602* on pSe-Kan. Position of the extra IS903 copy on pSBardo-Kan is indicated by an upside-down triangle on pSe-Kan plasmid. Open reading frames are marked by thick arrows; black, >90% protein identity to those of pSNC3-Kan; grey, <90% identity to those of pSNC3-Kan; white, no homologs on pSNC3-Kan. (B) Pairwise percent identity of KanR plasmids.

The pSNC3-Kan plasmid was determined to be 6,606 bps, 51.6% G+C. Sequence was deposited in NCBI GenBank under accession # MW030687. Plasmid features are listed in Table 2. It is comprised of RNA I/II region, *rom*, *mobC*-*mobA/BD* operon, and carried the “IS*903*-*aph(3’)-I*-IS*903* cassette” (Tn*602*; the two *tnp* genes arranged in the same orientation; [32, 33]), which is an indicative feature of the group C/C2 plasmids previously reported [20]. pSNC3-Kan was previously partially sequenced and assumed to be a variant of the group C plasmids due to their identical RNA I/II region and the *aph(3’)-I* gene, in addition to their similar restriction digest patterns. Yet to our surprise we found an operon of mobilization genes (*mobC* and *mobA/BD*), making the pSNC3-Kan backbone more closely resemble the typical ColE-like plasmids. Map of pSNC3-Kan and schematic comparison to other plasmids are shown in Fig 1.

**Table 2:** Plasmid pSNC3-Kan features.

Feature	Type	Coordinate	Putative function/ Notes
RNA I	misc_RNA	c129-238	RNA II inhibitor
RNA II	misc_RNA	127–670	Primer precursor
*oriV*	Origin	671–675	Origin of replication
*oriT* (ColE-type)	oriT	913–1001	Origin of transfer; based on pEC34B
*rom* (GTG start)	ORF	c1090-1281	RNA I modulator protein
*mobC*	ORF	1322–1669	Mobilization protein
*mobA*	ORF	1659–3212	Mobilization protein
*mobB*	ORF	2347–2865	Mobilization protein
*mobD* (GTG start)	ORF	2872–3105	Mobilization protein
Tn*602*	Mobile element	3543–6606	Transposon
IS*903-*a	Mobile element	3543–4599	IS element; IS*903* isoform IS*602*
*tnp*-IS*903-*a (TTG start)	ORF	c3599-4567	Transposase (IS*903*-a)
*aph(3’)*-I	ORF	c4618-5433	APH(3’)-I family aminoglycoside O-phosphotransferase; Kan^R^
IS*903-*b	Mobile element	5550–6606	IS element; IS*903* isoform IS*602*
*tnp*-IS*903-*b (TTG start)	ORF	c5606-6574	Transposase (IS*903*-b)

#### Tn*602*

The Tn*602* of pSNC3-Kan was 2-bp and 1-bp different from those of the pSe-Kan and pSBardo-Kan plasmids, respectively. The two IS*903* (isoform IS*602*) copies on pSNC3-Kan were 22-bp different from each other. The occurrence of Tn*602* is relatively rare and has been found on just 3 different plasmid backbones in nature: on IncFII plasmid pDG10, B/O plasmid R805a, and these KanR plasmids [32]. Upon re-examination of the pSe-Kan sequence, we identified the 9-bp target duplications “GTTGCTAAT” flanking the Tn*602* at coordinates 1–9 and 4060–4068; same target duplications were also found flanking the Tn*602* on pSBardo-Kan (nt 1–9 and 5126–5134). This was also pointed out by Moran and colleagues on their analysis of the Tn*602* on a large B/O plasmid R805a in *S*. Typhi [32]. However, there was only one such sequence in pSNC3-Kan at nt 1–9. It is conceivable that the Tn*602* was acquired by a common ancestor of the group C/C2/C3 plasmids possessing a *mob* operon, and then a recombination event resulted in the loss of the *mob* genes and replaced by the 3 ORFs of unknown function with low %G+C (~35%), resulting in a pSe-Kan-like plasmid. A BLASTN search (conducted on September 9, 2019) found one small plasmid in *E*. *coli* O111:H- strain 110512 (pO111-110512_6; AP019767) carrying the same 3 unknown ORFs with 99% identity to those of pSe-Kan and pSBardo-Kan; this was the only plasmid other than the group C/C2 KanR plasmids shown to possess such sequences.

#### RNA I/II and Rom

**Fig 2:**
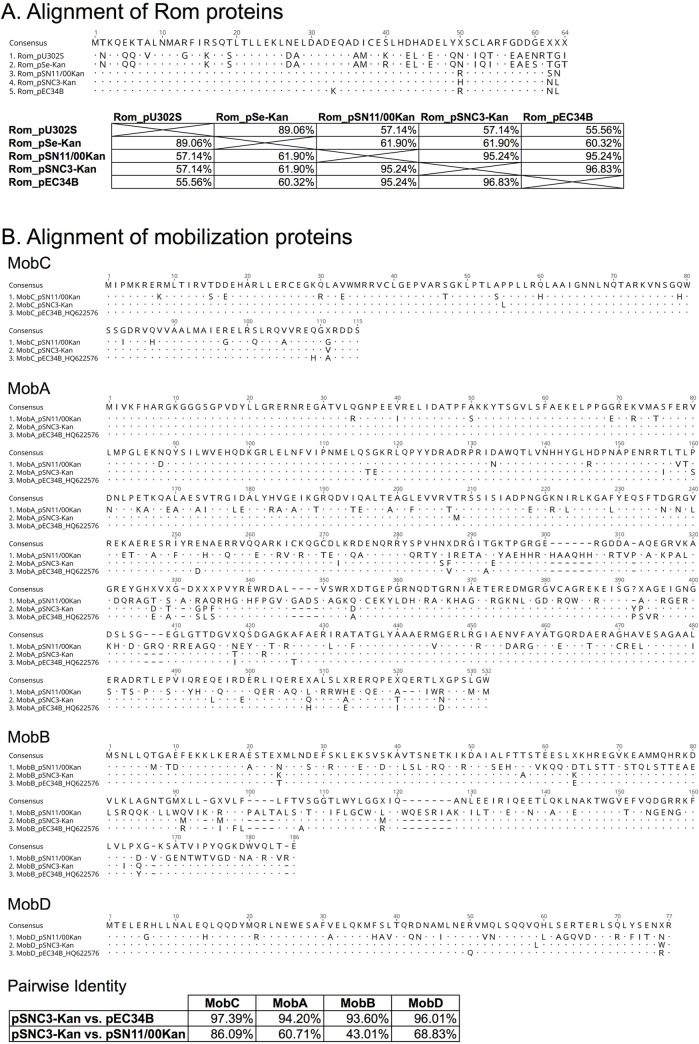
Alignment of Rom and mobilization proteins from pSNC3-Kan and other plasmids. (A) Alignment of Rom with pairwise identity table. (B) Alignment of mobilization proteins and pairwise identity table. Consensus is shown as the majority of the aligned sequences. “.” denotes identical amino acid sequence to that of the consensus. “–” indicates gap in alignment. “X” denotes not conserved residue (in consensus). “?” denotes the position with gap and not conserved residue (in consensus).

The RNA I/II region of pSNC3-Kan (nt 127–670) is 100% identical to those of group C/C2 plasmids pSe-Kan and pSBardo-Kan, and shared 98.6% and 97% identity to pU302S (group A) and pSN11/00Kan (group B), respectively. PlasmidFinder (v.2.1) did not identify any Col replicon using the default setting (95% identity/ 60% coverage). It was classified as a ColRNAI replicon only when the threshold settings were relaxed (89.6% to pIGMS32, coverage 125/130). Somewhat surprisingly, we found that the RNA I modulator protein (Rom) of pSNC3-Kan is more similar to that of the group B plasmid pSN11/00Kan (95.2% identity), than to the other group C/C2 plasmid pSe-Kan (61.9% identity). Alignment of Rom proteins are shown in Fig 2A. These findings agreed with the pairwise alignment that the sequence homology between pSNC3-Kan and C/C2 plasmids ends at nt 1038, about half-way between the first *oriT* and *rom* (as shown in Fig 1A).

Megablast against nr database found other plasmids with extremely high identity in the RNA I/II region, including *E*. *coli* plasmids pEC16II (99.1% identity/86.4% query coverage; KU932034; 7939-bp), and pEC34B (98.9% identity/100% query coverage; HQ622576; 6982-bp). Plasmid pEC34B from *E*. coli serotype O34 strain NCTC 9034 was conserved over the entire query length and well-annotated, thus was chosen for further comparison. Pairwise alignment of pSNC3-Kan against pEC34B showed homology spanning ~50% of the plasmid (>3.1-kb), from the *Bam*HI site (nt 83–88) extending to the end of the *mobA*, with 95.5% overall nucleotide identity; however, pSNC3-Kan did not possess a *mobE* gene downstream of *mobA*, as was the case in pEC34B (Fig 1A).

#### Mobilization proteins of pSNC3-Kan

Mobilization protein sequences from pSNC3-Kan, pSN11/00Kan, and pEC34B were aligned in Geneious (default setting: Global alignment with free end gap, cost matrix: Blosum62). The alignments and pairwise identity of the mobilization proteins MobC, MobA, MobB and MobD are shown in Fig 2B. Accessory DNA binding proteins containing the ribbon-helix-helix (RHH) domain(s) are involved in specific sequence recognition and binding near the *nic* site at *oriT* to help relaxase to form relaxosome and stimulate nickase function (for review, see [9, 15]). MobC, the mobilization accessory protein, of pSNC3-Kan was better conserved (86.09% identity) to that of pSN11/00Kan than the MobA relaxase and the internally encoded MobB and MobD (identity of 60.71%, 43.01%, and 68.83%, respectively); much greater protein homology (~95%) was observed to those of pEC34B. The N-terminal third (~155 residues) of the MobA proteins are highly conserved between those of pSN11/00Kan and pEC34B; the conserved Y19, SF and HQD-x4-ELNF relaxase motifs and residues [34] are all present in MobA_pSNC3-Kan. The low protein homology observed in the C-terminal two-thirds of the MobA, as well as MobB and MobD, between pSNC3-Kan and pSN11/00Kan strongly suggests that the *mob* region from these two plasmid groups were derived from different lineages.

Megablast of the *mobA* gene against nr database showed a considerable number of hits (94 of the 283) with >90% query coverage. The majority of those hits were in *E*. *coli*, with the maximum identity of 98% (100% query coverage) to an unannotated 7,991-bp *E*. *coli* plasmid p720632_7 (CP025843). The BLASTP top hits included proteins on *E*. coli plasmids pEC34B (96.6%; HQ622576), pLG3 (AF251289; 95.4%), and p5217 (NC_011799; 96.5%), although the max. identity was 100% (with 100% coverage) to proteins annotated as *E*. *coli* DNA polymerase (WP_047662141.1; KHH54811.1, KHH60013.1), or nuclease (MDL80525.1, MFR30254.1). The fact that pSNC3-Kan shares an extensive, conserved region spanning from RNA I/II to *mobC-mobA/BD* with the *E*. *coli* pEC34B plasmid, as well as having higher homologies to *E*. *coli* Mob proteins than to those of other *S*. *enterica* plasmids, may suggest that these two plasmids shared a recent common ancestor, and that pSNC3-Kan (and the group C ancestral plasmid) were recently acquired by *Salmonella* from *E*. *coli*. It is noteworthy that although the two plasmids are highly similar, the homology between pSNC3-Kan and pEC34B ended after the stop codon of *mobA*; the *mobE* gene was not present on pSNC3-Kan. The location of the potential XerC/XerD binding sites of the two plasmids were not conserved.

#### Origin of transfer (*oriT*)

**Fig 3:**
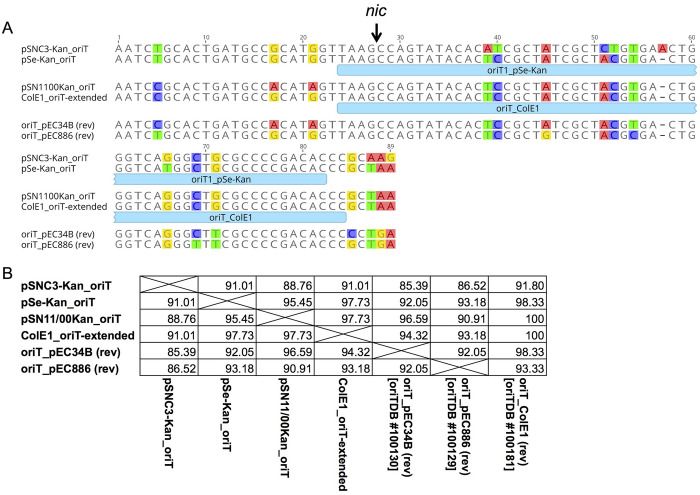
Comparison of *oriT* of pSNC3-Kan to other plasmids. (A) *oriT* sequence alignment. Non-identical bases are highlighted in color. Downward arrow indicates the *nic* site based on pEC34B annotation; the nicked strand is complementary to the sequence shown. (B) Pairwise percent identity table. Sequence labels started with “oriT” were retrieved from the *oriT*Finder database oriTDB and accession numbers are indicated within the square brackets in the pairwise comparison table. pSe-Kan *oriT* region was annotated based on the alignment to pEC34B, and is extended beyond the “oriT1_pSe-Kan” in oriTDB (shown as light blue box under the sequence). ColE1 sequence was extended to the same span as that of “oriT_pEC34B”; the “oriT_ColE1” in oriTDB is shown as light blue box under the sequence.

The *oriT* region was predicted to be at nt 913–1001 in pSNC3-Kan based on the annotation of pEC34B, although the *oriT*Finder identified a slightly shorter *oriT* at nt 912–997, with an *H*-value of 0.87 to that of pEC886 (*oriT*DB accession # 100129). The *oriT*Finder located the *oriT* on another KanR plasmid pSN11/00Kan at nt 989–1071 with a higher *H*-value of 0.93 to pEC34B (*oriT*DB accession # 100130). Sequence alignment and pairwise identity of the closely-related *oriT* regions of the KanR plasmids (excluding pU302S), plus those of the pEC34B, pEC886 and ColE1, are presented in Fig 3. Importantly, the putative 269-bp *oriT*(F-type) [labeled as “oriT2_pSe-Kan” in *oriT*DB, accession # 100060] found in the C/C2 plasmids (nt 1522–1790) was not identified in pSNC3-Kan.

#### Phylogenetic analysis of KanR plasmids

**Fig 4:**
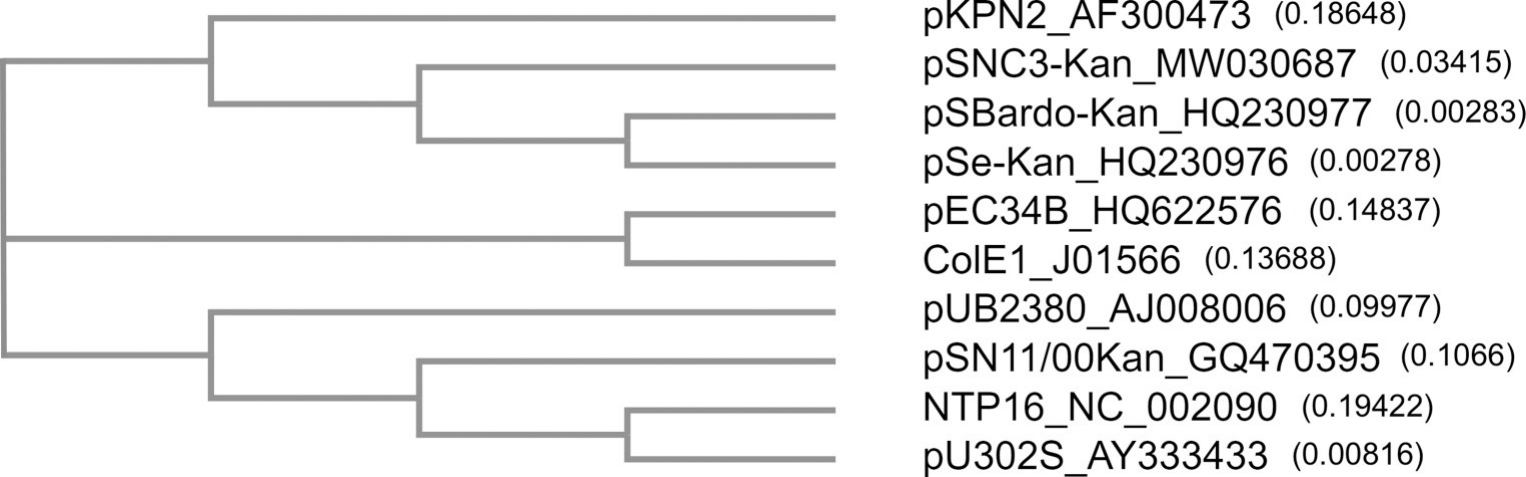
Cladogram of KanR plasmids and other plasmids. Neighbor-joining tree (without distance corrections) generated from multiple sequence alignment using MUSCLE. Plasmid name is followed by GenBank accession number (nucleotide sequence); branch length is indicated within the parentheses. Relevant information on the plasmids used in the alignment is listed in the S1 Table.

Multiple sequence alignment of nucleotide sequences of the five KanR plasmids, pKPN2 [35], ColE1 [36], pEC34B and two other related natural plasmids carrying *aph(3’)-I* (NTP16 [37] and pUB2380) was analyzed using MUSCLE and the resulting Neighbor-joining cladogram is shown in Fig 4. Information on the plasmids used in the alignment is listed in S1 Table. Group C/C2/C3 KanR plasmids were clustered on a separate branch from the group A and B plasmids pU302S and pSN11/00Kan, respectively. pKPN2, which showed an extended homologous region with the backbone of group C/C2/C3 KanR plasmids, also grouped appropriately. NTP16, possessing the *nikA* and *aph(3’)-I* gene, clustered with the *nikA*-bearing pU302S. pUB2380, a plasmid carrying *aph(3’)-I* and the *mob* region previously found in the BLASTN search of pSN11/00Kan [19], is on the same branch with pSN11/00Kan. pEC34B and ColE1 fell on a separate branch from the rest of the plasmids in this alignment, likely due to the differences in the cargo genes.

Plasmids are subject to continuous evolution/rearrangements: recombination, insertion/deletion, or acquiring genes from other mobile elements and chromosomes via transposons and insertion elements. This analysis is mainly to show the relatedness of the KanR plasmids, and does not necessarily imply the actual ancestry of these plasmids. The KanR plasmid grouping based on the restriction patterns corresponded well with the sequence comparison and phylogenetic relationship. The extremely high sequence identity of the three group C/C2/C3 plasmids strongly suggests that the divergence of these plasmids was a relatively recent event. On the other hand, these plasmids were distributed in several *S*. *enterica* serovars including Typhimurium (pSe-Kan), Bardo (pSBardo-Kan), Newport (pSNC3-Kan and pSe-Kan), Orion (pSe-Kan) and Brandenburg (pSNC3-Kan), isolated from cattle, dairy cattle or turkey [16, 17]. Although pSBardo-Kan was only isolated once, pSe-Kan and pSNC3-Kan were isolated multiple times from more than one serovar and different animal source, implicating the spread of these plasmids within *S*. *enterica*.

### Mobilization of KanR plasmids by *tra* genes of F and IncP plasmids

Mobilization of four representative KanR plasmids by the conjugal transfer (*tra*) genes derived from the F and IncP plasmid was evaluated using bi-parental mating. Many of the available IncP plasmids carried kanamycin resistance genes and thus could not be utilized. After an extensive search we opted to use the readily available, well-established S17-1λ*pir* strain to supply the required IncP *tra* genes. S17-1λ*pir* has the RP4-2 (*Km*::*Tn7*, *Tc*::*Mu-1*) plasmid integrated into the chromosome and is susceptible to Kan, Tet and Amp; the strain is commonly used to mobilize shuttle vectors via bi-parental mating [29]. Conjugal transfer genes of the F plasmid were supplied by the F’ plasmid in NEB5αF’I^q^. Results of the plasmid mobilization experiments are presented in Table 3. The S17-1λ*pir* donor-only controls showed ~2-fold increase after the 60-min incubation period, while NEB5αF’I^q^ donor-only control remained about the same (0.8- to 1.2-fold change). When no plasmid transfer occurred (indicated as “< 1x10^-7^” in Table 3), the total Kan-resistant counts were the same as the donor-only control counts. We evaluated the mobilization results based on the number of transconjugants in total kanamycin-resistant cells to simplify the experimental planning/logistics. This approach makes sense from the population perspective since the goal is to assess the contribution of plasmid transfer influencing the total antibiotic resistant population.

**Table 3:** Mobilization of KanR plasmids by conjugative transfer genes of IncP and F derivatives.

KanR plasmid		Donor × Recipient	
		S17-1λ*pir*	NEB5αF’I^q^
		×	×
		CAG18483	NEB10β
Name (KanR plasmid group)	Mobilization gene; *oriT*(type)	IncP *tra*	F *tra*
**pU302S (A)**	*nikA*;	<1 ×10^−7^^#^	1.47 ×10^−6^ ± 1.55 ×10^−6^
	*oriT*(pEC34A-type)		
**pSN11/00Kan (B)**	*mobC-mobA/BD*;	0.782 ± 0.099	<1 ×10^−7^
	*oriT*(ColE-type)		
**pSe-Kan (C)**	No known *mob* operon;	<1 ×10^−7^	0.687 ± 0.204
	*oriT*1(ColE-type), *oriT*(F-type)		
**pSNC3-Kan (C3)**	mobC-mobA/BD*;	0.690 ± 0.098	0.479 ± 0.522^$^
	*oriT*(ColE-type)		

*, *mobC-mobA/BD* of pSNC3-Kan are different from those of pSN11/00Kan.

^#^, no transconjugant was observed

^$^, average and standard deviation from 4 separate experiments (0.941, 0.922, 0.0373, 0.0172).

#### Mobilization by IncP *tra* genes

KanR plasmids possessing the ColE-type *oriT* and *mob* operons (pSN11/00Kan and pSNC3-Kan) were mobilized readily by the IncP *tra* genes, with over two-thirds of the Kan-resistant cells carrying mobilized KanR plasmid (Table 3). IncP *tra* genes could not mobilize pSe-Kan or pU302S, both without a *mob* operon. Although plasmids pSe-Kan and pSNC3-Kan share similar *oriT* (ColE-like) regions, pSe-Kan lacks the *mob* operon and no other *mob* genes were present in the donor cells, thus confirming that mobilization of these ColE-like plasmids by IncP *tra* genes require the mobilization accessory genes and a *cis*-acting *oriT*.

#### Mobilization by F *tra* genes

F *tra* genes mobilized plasmids pSNC3-Kan and pSe-Kan efficiently, but mobilization of pU302S occurred very infrequently (Table 3). High variability between experiments was observed for all 3 mobilizable KanR plasmids by F’, particularly for the pSNC3-Kan dataset which varied by as much as 55-fold (individual results were listed in the footnote of Table 3). F plasmid transfer was shown to be highly regulated and very sensitive to cell growth stage and environmental conditions- the transfer efficiency quickly declined after mid-exponential phase [38]. Although extra care was exercised to keep the OD600 of cultures between 0.3 and 0.5 when culturing the donor cells, we were not able to get maximal mobilization efficiency consistently.

**Fig 5:**
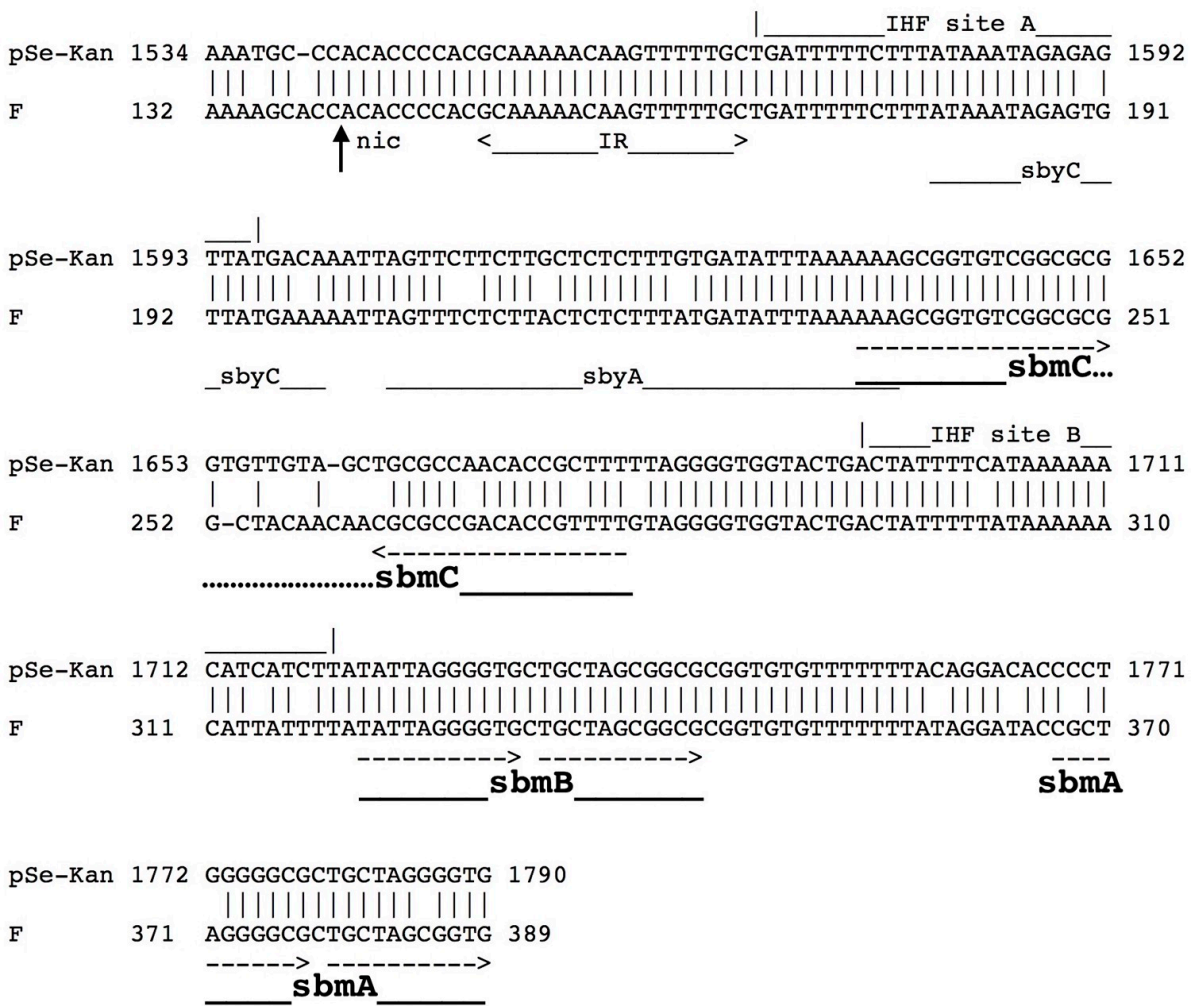
Alignment of *oriT(F)* of pSe-Kan to F. Relaxase cleavage site, nic, is marked by an upward arrow; the nicked strand is complementary to the sequence shown. IHF binding sites (A and B) are shown as lines above the alignment. TraM binding sites are marked by a dashed line with arrow. *SbmA/B/C*, TraM binding sites; sbyA/sbyC, TraY binding sites. Sites were marked based on [40, 41].

ColE1 derivatives can be mobilized by various conjugative plasmids, especially by IncFI (F’*lac pro*) very efficiently; this requires the presence of the *cis*-acting *oriT* (*bom*) region and mobilization proteins on the same or other complementing plasmids [10]. The fact that pSe-Kan lacked a *mob* operon but was still mobilizable by F *tra* genes, suggests that the *oriT(F)* may be sufficient to facilitate the binding of the F conjugal transfer proteins without the assistance of other mobilization relaxase and accessory proteins- a scenario of “relaxase-*in-trans*” [39]. The *oriT(F)* of pSe-Kan is ~90% identical to the F *oriT* (U01159.2; [40]). The alignment and the binding sites of TraM, TraY, and the Integration host factor (IHF) are shown in Fig 5. The F TraY accessory protein causes DNA bending when bound to *sby* sites and stimulates the relaxase activity of TraI at the *nic* site; IHF-mediated DNA bending also alters the ability of relaxases to melt and cleave DNA. While TraM is not required for cleavage, it is important for conjugation, likely through interaction with the TraD coupling protein to move the relaxosome to the conjugative pore [9, 15, 41]. The two high affinity TraM binding sites *sbmA* and *sbmB* assist and stabilize TraM binding to the lowest affinity site *sbmC*, which is critical for conjugation [41]. Hydroxyl radical footprinting [41] showed two TraM dimer footprints centered at the ACAACA sequence between the inverted repeats at *sbmC*, where the least conserved region between pSe-Kan and F is located. Although there was no sequence conservation at the *sbmC* contact sites [41], the significance of these mismatches cannot be ascertained without further research. The *oriT(F)* of pSe-Kan is 100% identical to another small plasmid pKPN2, which also lacks *mob* genes (AF300473; [36]). pKPN2 shared high sequence identity (95%) with pSe-Kan in the ~2-kb region (nt 81–2039) spanning from RNA I/II to *cer*, which includes the identical *oriT(F)* region, and 1-bp mismatch in *oriT1*(ColE-type) (alignment shown in S1 Fig). pKPN2 was shown to be mobilized by F’(*lac-gal*) plasmid with high efficiency [36]. When we examined the plasmid minipreps from the transconjugants, the mobilized KanR plasmids were observed in all the plasmid preps. Taken together the highly conserved sequence and similar mobilization results of pKPN2, we think that this further supports our finding that the pSe-Kan plasmid mobilization by F *tra* genes does not require the plasmid-borne mobilization proteins. However, the possibility that pSe-Kan might form cointegrates with the F’ plasmid cannot be completely ruled out.

On the other hand, the failure to mobilize pSN11/00Kan by F *tra* genes was somewhat puzzling, given that the plasmid possesses a highly conserved *oriT* to ColE1 (Fig 3) and moderately conserved *mobC-mobA/BD* operon to those of pSNC3-Kan (Fig 2). The *nic* site within *oriT*, the relaxase, and accessory proteins all contribute to the transfer specificity [9]. It is possible that the interaction between the pSN11/00Kan mobilization proteins, *oriT*, and the F conjugal transfer proteins was not optimal, and may favor other conjugative plasmids such as IncP (see above) or IncI. Further in-depth studies are needed to reveal the precise molecular mechanism(s) involved in the observed differences in mobilization of different KanR plasmids by the *tra* genes of different conjugative plasmids.

Mobilization of pU302S (group A) by F’ was only detected at a very low occurrence. pU302S was present in all the plasmid preps from the transconjugants. It is unlikely that cointegrates were formed between pU302S and F’, since a higher transfer efficiency would have been expected. The 108-bp *oriT* sequence on pU302S is that of the pEC34A-type (*oriT*DB accession #100131; H-value = 0.97), shared ~78% identity to the *oriT* of IncI1 plasmid R64 but with longer (20-bp) inverted repeats [14]. There was no significant similarity found to the F *oriT* using BLASTN (Expect value 0.05). Moran and Hall analyzed pCERC7 and related *nikA*-bearing small plasmids, including pU302S and NTP16, and showed that all carried identical NikA (53% identity to that of R64), and extremely conserved *oriT* (up to 3 mismatches); none of these plasmids carried the NikB relaxase homolog [14]. NikA protein is expected to bind to the inverted repeats in the *oriT* and interact with the relaxase of an I-complex plasmid to initiate transfer [14]. NTP16 was the only plasmid in this *nikA*-bearing family that has been shown to be mobilized by R64 [14, 37], suggesting that its NikA can interact with the relaxase of R64. The homology of NikA and *oriT* of this *nikA*-bearing plasmid family to the IncI1 system is moderate, it is plausible that another yet-to-be-identified conjugative plasmid group may interact with this plasmid family more efficiently. Alternatively, the system may have evolved to be more flexible to work with multiple conjugative systems. Mobilization of this *nikA*-bearing family by F has never been reported. Although the structure of the pU302S/NTP16 NikA protein has not been determined, it is reasonable to assume that it retains the highly conserved RHH domain structure of NikA_R64 and MbeC_ColE1, as shown by Varsaki and colleagues [34]. Since MbeC_ColE1 can interact with many different conjugative T4SS including those of F and I1, based on our experimental findings we speculate that NikA_pU302S may interact with the conjugative system of F, albeit less efficiently, to initiate transfer. To our knowledge this is the first report of a plasmid bearing *nikA* to be mobilized by F *tra* genes. Since F plasmid transfer is sensitive to growth stage, the log phase cultures utilized in our mating experiments may have played a role in optimizing these inefficient mobilization events.

## Discussion

Although the original *S*. Newport isolate ARS# 574 was shown to carry additional IncA/C and I1 replicons and no co-resident IncF or P plasmids [16], other KanR plasmids came from *S*. *enterica* isolates carrying IncFI/FII, P, I1, or other replicons [16, 18–20, 42]. We recently isolated and sequenced several conjugative plasmids belonging to the IncI1, X1 and X4 groups that were able to mobilize at least some of the KanR plasmids using a qualitative tri-parental mating assay [43]. Although not reported in that study, pSNC3-Kan was mobilizable by all the conjugative IncI1, X1 and X4 plasmids identified, in a pattern similar to that of pSN11/00Kan; on the other hand, pU302S and pSe-Kan were mobilizable only by IncI1 plasmids, but not by IncX1 or X4 plasmids [43]. No IncA/C plasmid was identified in the same study [43]. Other attempts to acquire or isolate IncA/C plasmids capable of mobilizing KanR plasmids were also unsuccessful because all available IncA/C plasmids or strains were also resistant to kanamycin.

Ares-Arroyo and colleagues have shown that 74% of the Enterobacteriaceae isolates tested carried at least one ColE-like plasmid using a PCR-based system for detection and capture of plasmid sequences [44]. Cohen and colleagues [45] detected small plasmids (2.2 to 6 kb) in 17 out of 19 MDR *S*. *enterica* poultry isolates of a variety of serovars in Israel; some of these also carried a co-resident IncI1 or X1 conjugative plasmid. Among these, a Newport isolate was shown to harbor 3 small plasmids (2.2, 3.2, and 6 kb) and a 44.6-kb IncX1 plasmid; however, mobilization of these small plasmids was not tested [45]. A recent study by Oliva and colleagues [46] showed that two small ColE-like plasmids in a *S*. Typhimurium MDR strain ST1030 were co-transferred with the large IncI1 conjugative plasmid pST1030-1A at very high frequencies: pST1030-2A (possessed *mobC-mobABDE*) was found in 92% of the transconjugants and ~56% of the transconjugants harbored both small plasmids pST1030-2A and pST1030-3 (which possessed an orphan *mob*-associated *oriT*) [39, 46]. Another example of “relaxase-*in-trans*” was reported by Moran and Hall [47]: a 1982-bp cryptic rolling-circle plasmid, pBuzz, which carried two copies of *oriT*’s but without the relaxase or other accessory genes, was mobilized by the co-resident B/O plasmid with high efficiency; other related rolling-circle plasmids may also carry diverse *oriT*s of different Inc types [47]. These studies, as well as ours, showed that numerous variations exist in the mobilization mechanisms/strategies employed by the small plasmids, many of which do not require a plasmid-borne mobilization relaxase protein. We concur with the notion that the approach using relaxase alone to predict potential plasmid transferability is insufficient, and underestimates the influence of small plasmids in horizontal gene transfer [39, 47].

## Concluding remarks

Here we demonstrated that ColE-like KanR plasmids can be mobilized with varying efficiency by conjugative plasmids F or IncP in *E*. *coli*. Plasmid pSNC3-Kan was the only KanR plasmid that can be mobilized by both F and IncP *tra* genes. We showed that the F conjugative system is capable of mobilizing pSe-Kan which lacks the mobilizable plasmid-borne relaxase or accessory proteins, likely through the interaction with *oriT(F)*. We also reported the first known case of *nikA*-bearing plasmid mobilized by F (at low efficiency). However, without mechanistic proof we can only speculate that the NikA and *oriT* of pU302S may be able to interact with the F *tra* genes. These naturally occurring kanamycin resistance plasmids with different mobilization accessory proteins and *oriT* regions are valuable resources in providing insights on how small plasmids are mobilized by different conjugative plasmids. Although studies on the identification and sequencing of small plasmids have increased significantly due to a large number of whole genome sequencing projects, comprehensive annotation of the small plasmids is often lacking. Research on the capability and mechanisms of small plasmid mobilization is still extremely limited and deserves more attention.

## Supporting information

S1 Table Plasmid information used in the multiple sequence alignment of Fig 4. (DOCX)

S1 Fig Sequence alignment of pSe-Kan and pKPN2. Plasmid sequences were aligned using BLASTN (Align two or more sequences). Multiple sequence alignment viewer image was shown below the alignment.
(PDF)
