# Supplementary material for: Sequence analysis and plasmid mobilization of a 6.6-kb kanamycin resistance plasmid, pSNC3-Kan, from a Salmonella enterica serotype Newport isolate
Source: PLoS One. 2022 Jul 14;17(7):e0268502. doi: 10.1371/journal.pone.0268502 (PMC9282650; doi:10.1371/journal.pone.0268502)
Supplement: S1 Table — (DOCX) [file pone.0268502.s001.docx]

**S1 Table. Plasmid information used in the multiple sequence alignment of Fig 4.**

| **Plasmid** | **KanR group** | **Size (bp)** | ***aph(3')-I*** | **mobilization gene(s)** | **Bacteria** | **Reference** |
| --- | --- | --- | --- | --- | --- | --- |
| pKPN2 |  | 4196 | – | – | *Klebsiella pneumoniae* | [35] |
| pSNC3-Kan | C3 | 6606 | + | *mob* operon | *Salmonella enterica* serovar Newport | this study |
| pSBardo-Kan | C2 | 8198 | + | – | *Salmonella enterica* serovar Bardo | [20] |
| pSe-Kan | C | 7132 | + | – | *Salmonella enterica* serovar Typhimurium | [20] |
| pEC34B |  | 6982 | – | *mob* operon | *Escherichia coli* serotype O34 | direct submission |
| ColE1 |  | 6646 | – | *mob* operon | *Escherichia coli* | [36] |
| pUB2380 |  | 8561 | – | *mob* operon | *Escherichia coli* | direct submission |
| pSN11/00Kan | B | 5698 | + | *mob* operon | *Salmonella enterica* serovar Newport | [19] |
| NTP16 |  | 8255 | + | *nikA* | *Salmonella enterica* serovar Typhimurium | [37] |
| pU302S | A | 3208 | + | *nikA* | *Salmonella enterica* serovar Typhimurium | [18] |
