# Supplementary material for: Sequence analysis and plasmid mobilization of a 6.6-kb kanamycin resistance plasmid, pSNC3-Kan, from a Salmonella enterica serotype Newport isolate
Source: PLoS One. 2022 Jul 14;17(7):e0268502. doi: 10.1371/journal.pone.0268502 (PMC9282650; doi:10.1371/journal.pone.0268502)

Query: Salmonella enterica subsp. enterica serovar Typhimurium plasmid pSe-Kan, complete sequence Query ID: HQ230976.1 Length: 7132

Subject: Klebsiella pneumoniae plasmid pKPN2, complete sequence
Sequence ID: AF300473.1 Length: 4196
Range 1: 9 to 1976

Score:3110 bits(1684), Expect:0.0,
Identities:1883/1976(95%), Gaps:25/1976(1%), Strand: Plus/Plus

```
Query  81    AAGGATCCTCTTGTGATCCtttttttCTGCGCGTAATCTGCTGCCTGTAAACaaaaaaaC  140
             |||||| |||||| ||||||||||||||||||||||||||    ||||||||| |||||||
Sbjct  9     AAGGAT-CTCTTGAGATCCTTTTTTTCTGCGCGTAATCTTGGACCTGTAAACGAAAAAAC  67

Query  141   CACCGCTACCAACGGTGGTTTGTTTGCCGG-AATCTAGAGCTACCAACTCTTTCT-TCCG  198
             |||| ||  | | |||||||| || |  ||  | |||   ||  ||  | | |||  |||
Sbjct  68    CACC-CTGGC-A-GGTGGTTTTTTCGAAGGTTAGCTAATCCTGGCAGAT-TATCTAACCG  123

Query  199   AAGGTAA-CTGGCTT-GGTGGAGCACAGATACCAAATACTGTCCTTCTAGTGTAGCCGCA  256
              |||||| |||||||  |  ||||||||||||||||||||||||||| |||||||||| |
Sbjct  124   -AGGTAATCTGGCTTCAGCAGAGCACAGATACCAAATACTGTCCTTCCAGTGTAGCCGTA  182

Query  257   GTTAGGCCACCACTTCAAGAACTCT-TAA-TATC----TCAATCCAC-CT-TG----TCC  304
             ||||||||| ||||||||||||||| |||  |||    | ||||| | || ||    |||
Sbjct  183   GTTAGGCCATCACTTCAAGAACTCTGTAAGCATCTGGATAAATCCTCGCTCTGCTAATCC  242

Query  305   AGTTACCAGTGGCTGCTGCCAGTGGCGCTTTGTCGTGTCTTACCGGGTTGGACTCAAGAC  364
              |||||||||||||||||||||||||| |  | |||||||||| ||||||||||||||||
Sbjct  243   GGTTACCAGTGGCTGCTGCCAGTGGCGTTAAGGCGTGTCTTACTGGGTTGGACTCAAGAC  302

Query  365   GATAGTTACCGGATAAGGCGCAGCGGTCGGGCTGAACGGGGGGTTCGTGCACACAGCCCA  424
             ||||||||||||||||||||||||||||||||||||||||||||||||||||||||||||
Sbjct  303   GATAGTTACCGGATAAGGCGCAGCGGTCGGGCTGAACGGGGGGTTCGTGCACACAGCCCA  362

Query  425   GCTTGGAGCGAACGACCTACACCGAACTGAGATACCAACAGCGTGAGCTATGAGAAAGCG  484
             ||||||||||||||||||||||||  | ||||||||||||||||||||||||||||||||
Sbjct  363   GCTTGGAGCGAACGACCTACACCGGGCCGAGATACCAACAGCGTGAGCTATGAGAAAGCG  422

Query  485   CCACGCTTCCCGAAGGGAGAAAGGCGGACAGGTATCCGGTAAGCGGCAGGGTCGGAACAG  544
             ||||||||||||||||||||||||||||||||||||||||||||||||||||||||||||
Sbjct  423   CCACGCTTCCCGAAGGGAGAAAGGCGGACAGGTATCCGGTAAGCGGCAGGGTCGGAACAG  482

Query  545   GAGAGCGCACGAGGGAGCTTCCAGGGGGAAACGCCTGGTATCTTTATAGTCCTGTCGGGT  604
             ||||||||||||||||||||||||||||||||||||||||||||||||||||||||||||
Sbjct  483   GAGAGCGCACGAGGGAGCTTCCAGGGGGAAACGCCTGGTATCTTTATAGTCCTGTCGGGT  542

Query  605   TTCGCCACCTCTGACTTGAGCGTCGATTTTTGTGATGCTCGTCAGGGGGGCGGAGCCTAT  664
             ||||||||| ||||||||||||||||||||||||||||||||||||||||||||||||||
Sbjct  543   TTCGCCACCCCTGACTTGAGCGTCGATTTTTGTGATGCTCGTCAGGGGGGCGGAGCCTAT  602

Query  665   GGAAAAACGCCATCGGCGTGGCCTTTCTCCCGGCCTTTGCC-TTTTGCGCACATGTTCTT  723
             |||||||||||| ||||| ||||||| |   | |||||||| |||| |||||||||||||
Sbjct  603   GGAAAAACGCCAGCGGCGCGGCCTTT-TGTTGCCCTTTGCCTTTTTACGCACATGTTCTT  661
```

```
Query  724   TCCGGCGTTATCCCCTGATTCTGTGGATAACCGTATTACCGCCTTTGAGTGAGCTGATAC  783
             |||||| |||||||||||||||||||||||||||||| |||||||||||||||| |  ||
Sbjct  662   TCCGGCCTTATCCCCTGATTCTGTGGATAACCGTATTTCCGCCTTTGAGTGAGCCGGCAC  721

Query  784   CGCTCGCCGCAGCCGAACGACCGAGCGTAGCGAGTCAGTGAGCGAGGAAGCGGAAGAGCG  843
             |||| |||||||||||||||||||||| ||||||||||||||||||||||||||||||||
Sbjct  722   CGCTTGCCGCAGCCGAACGACCGAGCGCAGCGAGTCAGTGAGCGAGGAAGCGGAAGAGCG  781

Query  844   TCGGAAGCTGCATTTTCTCCTTACGCATCTGTGCGGCATTTCACACCCGGCATGGCGCAC  903
              | || || | || |||||||||||||||||||||||||||||||||||||||||||  |
Sbjct  782   CCTGATGCGGTATGTTCTCCTTACGCATCTGTGCGGCATTTCACACCCGGCATGGCGTGC  841

Query  904   TTTTCATACAATCTGCACTGATGCCGCATGGTTAAGCCAGTATACACTCCGCTATCGCTA  963
             ||||||||||||| ||||||||||||||| ||||||||||||||||||||||||||||||
Sbjct  842   TTTTCATACAATCCGCACTGATGCCGCATAGTTAAGCCAGTATACACTCCGCTATCGCTA  901

Query  964   CGTGACTGGGTCATGGCTGCGCCCCGACACCCGCTAAAACCCCCTGACGCGCCCTGACGG  1023
             ||||||||||||||||||||||||||||||||||||||||||||||||||||||||||||
Sbjct  902   CGTGACTGGGTCATGGCTGCGCCCCGACACCCGCTAAAACCCCCTGACGCGCCCTGACGG  961

Query  1024  GCTTGTCTGCTGACGAAAACGCCCACGGGAAAGCCGTGACCGCCTCCGTCAGCTTCGTTA  1083
             ||||||||||||||||||||||||||||||||||||||||||||||||||||||||||||
Sbjct  962   GCTTGTCTGCTGACGAAAACGCCCACGGGAAAGCCGTGACCGCCTCCGTCAGCTTCGTTA  1021

Query  1084  TGTTCCTGTTTCACTTTCGGCCTCAAAGCGAATCTGGATGCTGTTCTGGAGTTCTTCTGC  1143
             ||||||||||||||||||||||||||||||||||||||||||||||||||||||||||||
Sbjct  1022  TGTTCCTGTTTCACTTTCGGCCTCAAAGCGAATCTGGATGCTGTTCTGGAGTTCTTCTGC  1081

Query  1144  GAGTTCGTGCAGTCGTTCGCACATGGCCGCCTGTTCGTCGGCATCCAGAGCATCCAGTTT  1203
             ||||||||||||||||||||||||||||||||||||||||||||||||||||||||||||
Sbjct  1082  GAGTTCGTGCAGTCGTTCGCACATGGCCGCCTGTTCGTCGGCATCCAGAGCATCCAGTTT  1141

Query  1204  TTCAAGCAGTGTCAGGCTCTGGCTTTTGATAAATCGCGCCATATTCAGCGCAGTTTGTTG  1263
             ||||||||||||||||||||||||||||||||||||||||||||||||||||||||||||
Sbjct  1142  TTCAAGCAGTGTCAGGCTCTGGCTTTTGATAAATCGCGCCATATTCAGCGCAGTTTGTTG  1201

Query  1264  TTGTTTGTTCACCTTCCGTTGTTCTCCGTCAGTTCCGGGTGTCGGGGGGACCCTGAGCAG  1323
             ||||||||||||||||| ||||||||||||||||||||||||||||||||||||||||||
Sbjct  1202  TTGTTTGTTCACCTTCCTTTGTTCTCCGTCAGTTCCGGGTGTCGGGGGGACCCTGAGCAG  1261

Query  1324  GTGGTAATTGTAAGACATTGCGGGCGACGGTATTACAATTGCACATCCTGTCCTGTTTCT  1383
             |||||||||||||||| |||||||||||||||||||||||||||||||||||||||||||
Sbjct  1262  GTGGTAATTGTAAGACGTTGCGGGCGACGGTATTACAATTGCACATCCTGTCCTGTTTCT  1321

Query  1384  TTAGGACGTATCATGGCACAGCATATCAACTGATAAATAATCCCTGTGCAGTGCAGCCGC  1443
             ||||||||||||||||||||||||||||||||||||||||||||||||||||||||||||
Sbjct  1322  TTAGGACGTATCATGGCACAGCATATCAACTGATAAATAATCCCTGTGCAGTGCAGCCGC  1381

Query  1444  TGTTGCATAAAATTTCGCTCTGCGTCGAGTGTTTCTTAATCAATAATTCCGCTGATACTC  1503
             ||||||||||||||||||||||||||||||||||||||||||||||||||||||||||||
Sbjct  1382  TGTTGCATAAAATTTCGCTCTGCGTCGAGTGTTTCTTAATCAATAATTCCGCTGATACTC  1441
```

```
Query  1504  CGGAAAAACGCGAGTCTGTTCTCATGCCTGAAATGCCCACACCCCACGCAAAAACAAGTT  1563
             ||||||||||||||||||||||||||||||||||||||||||||||||||||||||||||
Sbjct  1442  CGGAAAAACGCGAGTCTGTTCTCATGCCTGAAATGCCCACACCCCACGCAAAAACAAGTT  1501

Query  1564  TTTGCTGATTTTTCTTTATAAATAGAGAGTTATGACAAATTAGTTCTTCTTGCTCTCTTT  1623
             ||||||||||||||||||||||||||||||||||||||||||||||||||||||||||||
Sbjct  1502  TTTGCTGATTTTTCTTTATAAATAGAGAGTTATGACAAATTAGTTCTTCTTGCTCTCTTT  1561

Query  1624  GTGATATTTAAAAAAAGCGGTGTCGGCGCGGTGTTGTAGCTGCGCCAACACCGCTTTTTAG  1683
             ||||||||||||||||||||||||||||||||||||||||||||||||||||||||||||
Sbjct  1562  GTGATATTTAAAAAAAGCGGTGTCGGCGCGGTGTTGTAGCTGCGCCAACACCGCTTTTTAG  1621

Query  1684  GGGTGGTACTGACTATTTTCATAAAAAACATCATCTTATATTAGGGGTGCTGCTAGCGGC  1743
             ||||||||||||||||||||||||||||||||||||||||||||||||||||||||||||
Sbjct  1622  GGGTGGTACTGACTATTTTCATAAAAAACATCATCTTATATTAGGGGTGCTGCTAGCGGC  1681

Query  1744  GCGGTGTGtttttttACAGGACACCCCTGGGGGCGCTGCTAGGGGTGTCTGTTCAGATAT  1803
             ||||||||||||||||||||||||||||||||||||||||||||||||||||||||||||
Sbjct  1682  GCGGTGTGTTTTTTTACAGGACACCCCTGGGGGCGCTGCTAGGGGTGTCTGTTCAGATAT  1741

Query  1804  GTAGCAATGAGTCAGGGATTCACGAACTGAAAATGTCCGAAAATGTGTCTCATGGGCACT  1863
             ||||||||||||||||||||||||||||||||||||||||||||||||||||||||||||
Sbjct  1742  GTAGCAATGAGTCAGGGATTCACGAACTGAAAATGTCCGAAAATGTGTCTCATGGGCACT  1801

Query  1864  GTATGCGCCCAAACCGATCGTCATTCACTTTCATGCATAGCTATGCAGTGAGCTGAAAGC  1923
             ||||||||||||||||||||||||||||||||||||||||||||||||||||||||||||
Sbjct  1802  GTATGCGCCCAAACCGATCGTCATTCACTTTCATGCATAGCTATGCAGTGAGCTGAAAGC  1861

Query  1924  GATCCTGACGCATTTTTCCGGTTTACCCCGGGGAGAACATCTCTTTTTGCGGTGTTCGCG  1983
             ||||||||||||||||||||||||||||||||||||||||||||||||||||||||||||
Sbjct  1862  GATCCTGACGCATTTTTCCGGTTTACCCCGGGGAGAACATCTCTTTTTGCGGTGTTCGCG  1921

Query  1984  GCAGAATCGCTTTCAGCGCGTTTTAGCGGTGCGCGCAATGCGACGTTATGGTAAAT  2039
             ||||||| ||||||||||||||||||||||||||||||||||||||||||||||||
Sbjct  1922  GCAGAAT-GCTTTCAGCGCGTTTTAGCGGTGCGCGCAATGCGACGTTATGGTAAAT  1976
```

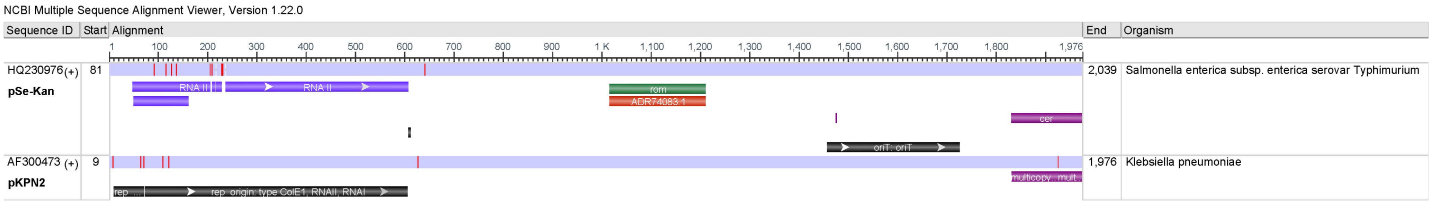

Supplement: S1 Fig — Plasmid sequences were aligned using BLASTN (Align two or more sequences). Multiple sequence alignment viewer image was shown below the alignment. (PDF) [file pone.0268502.s002.pdf]
